# Mapping of sentinel lymph node drainage using SPECT/CT to tailor elective nodal irradiation in head and neck cancer patients (SUSPECT-2): a single-center prospective trial

**DOI:** 10.1186/s12885-019-6331-8

**Published:** 2019-11-14

**Authors:** Pieter D. de Veij Mestdagh, Willem H. Schreuder, Wouter V. Vogel, Maarten L. Donswijk, Eric van Werkhoven, Jacqueline E. van der Wal, Richard Dirven, Baris Karakullukcu, Jan-Jakob Sonke, Michiel W. M. van den Brekel, Corrie A. M. Marijnen, Abrahim Al-Mamgani

**Affiliations:** 1 Department of Radiation Oncology, The Netherlands Cancer Institute, Plesmanlaan 121, 1066 CX Amsterdam, The Netherlands; 2 Department of Head and Neck Surgery, The Netherlands Cancer Institute, Amsterdam, The Netherlands; 3 Department of Nuclear Medicine, The Netherlands Cancer Institute, Amsterdam, The Netherlands; 4 Department of Biometrics, The Netherlands Cancer Institute, Amsterdam, the Netherlands; 5 Department of Pathology, The Netherlands Cancer Institute, Amsterdam, the Netherlands

**Keywords:** Head and neck cancer, Unilateral elective irradiation, Bilateral elective irradiation, Lymph drainage mapping, Sentinel node

## Abstract

**Background:**

The majority of patients with head and neck squamous cell carcinoma (HNSCC) receive bilateral elective nodal irradiation (ENI), in order to reduce the risk of regional failure. Bilateral ENI, as compared to unilateral ENI, is associated with higher incidence of acute and late radiation-induced toxicity with subsequent deterioration of quality of life. Increasing evidence that the incidence of contralateral regional failure (cRF) in lateralized HNSCC is very low (< 10%) suggests that it can be justified to treat selected patients unilaterally. This trial aims to minimize the proportion of patients that undergo bilateral ENI, by using lymph drainage mapping by SPECT/CT to select patients with a minimal risk of contralateral nodal failure for unilateral elective nodal irradiation.

**Methods:**

In this one-armed, single-center prospective trial, patients with primary T1-4 N0-2b HNSCC of the oral cavity, oropharynx, larynx (except T1 glottic) or hypopharynx, not extending beyond the midline and planned for primary (chemo) radiotherapy, are eligible. After ^99m^Tc-nanocolloid tracer injection in and around the tumor, lymphatic drainage is visualized using SPECT/CT. In case of contralateral lymph drainage, a contralateral sentinel node procedure is performed on the same day. Patients without contralateral lymph drainage, and patients with contralateral drainage but without pathologic involvement of any removed contralateral sentinel nodes, receive unilateral ENI. Only when tumor cells are found in a contralateral sentinel node the patient will be treated with bilateral ENI. The primary endpoint is cumulative incidence of cRF at 1 and 2 years after treatment. Secondary endpoints are radiation-related toxicity and quality of life. The removed lymph nodes will be studied to determine the prevalence of occult metastatic disease in contralateral sentinel nodes.

**Discussion:**

This single-center prospective trial aims to reduce the incidence and duration of radiation-related toxicities and improve quality of life of HNSCC patients, by using lymph drainage mapping by SPECT/CT to select patients with a minimal risk of contralateral nodal failure for unilateral elective nodal irradiation.

**Trial registration:**

ClinicalTrials.gov Identifier: NCT03968679, date of registration: May 30, 2019.

## Background

The great majority of patients with head and neck squamous cell carcinoma (HNSCC) receive elective nodal irradiation (ENI) to both sides of the neck in order to reduce the risk of contralateral regional failure (cRF). However, there is increasing evidence that the incidence of cRF in lateralized HNSCC is very low (< 10%) [1–5]. Bilateral ENI, as compared to unilateral ENI, is associated with higher incidence of acute and late radiation-induced toxicity with subsequent deterioration of quality-of-life (QoL) [6–11]. One way to reduce the incidence and severity of these toxicities is by implementation of unilateral ENI, in patients where this can be justified.

**Fig. 1:**
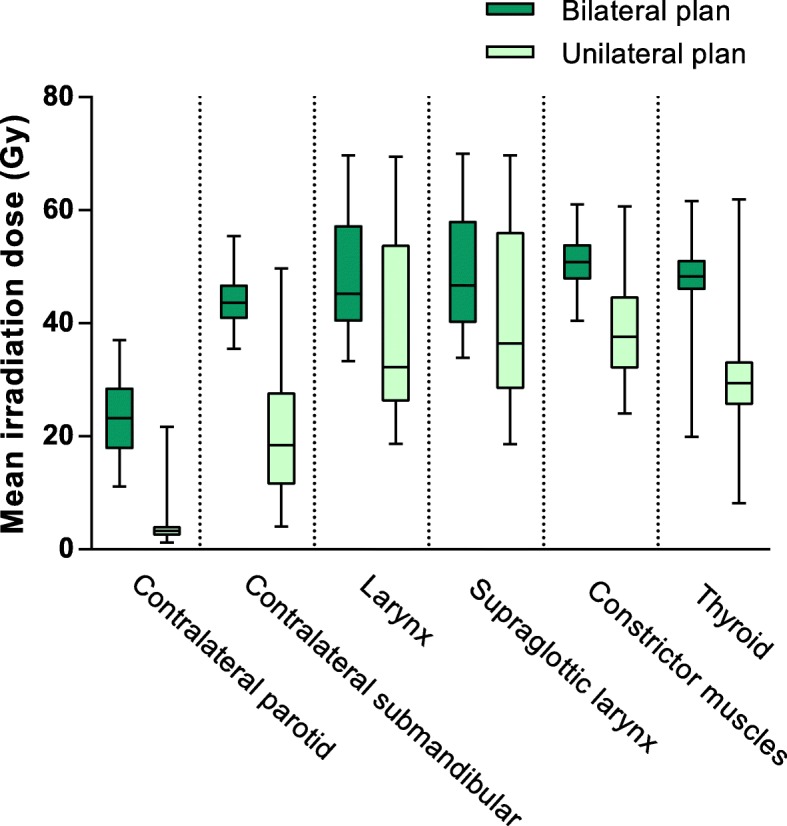
Dose reduction to organs at risk. Boxplot of planned mean irradiation doses to organs at risk. For every patient treated within the SUSPECT-1 study, two plans were made: a unilateral plan based on the results of the SPECT/CT, that was used to treat the patient, and (for comparison purposes) a regular bilateral plan they would have been treated with outside the framework of the study. The mean doses to all these organs at risk were significantly lower in the unilateral plan, compared to the regular bilateral plan. Abbreviation: Gy: gray

The first SUSPECT study (ClinicalTrials.gov Identifier NCT02572661) investigated whether lymph drainage mapping (LDM) using Single Photon Emission Computed Tomography/Computed Tomography (SPECT/CT) was a safe and feasible method to exclude the contralateral neck from the elective irradiation fields, or, in case of contralateral lymph drainage, to tailor the contralateral ENI field only to the level containing the tracer accumulation [12, 13]. Large dose reductions to most organs at risk were realized (Fig. 1). Moreover, we found significant reductions of both short term (mucositis, dysphagia) and long term (xerostomia, dysphagia) toxicities (manuscript in preparation).

Since around 20% of patients treated within the first SUSPECT study had contralateral tracer accumulation (usually in one neck level), a substantial proportion of patients still received an elective irradiation dose to one contralateral neck level. In studies investigating sentinel node procedures (SNP) for oropharynx and oral cavity carcinoma, contralateral sentinel nodes (SNs) were found in 8–40% of patients. However, the overall prevalence of tumor-positive contralateral SNs was only 0–2.5% [2, 4, 14, 15]. Some studies suggest the overall prevalence might be higher (around 10%) in larynx and hypopharynx carcinoma [5, 16]. Even so, elective irradiation to all contralateral draining sentinel nodes might still be overtreatment.

The aim of the current manuscript is to introduce the sequel to this study, the SUSPECT-2 study (ClinicalTrials.gov Identifier NCT03968679). Like the first SUSPECT study, the SUSPECT-2 study selects patients for unilateral treatment if they have no contralateral lymphatic drainage from the tumor site, but it modifies the original concept by performing a contralateral SNP in case of contralateral lymph drainage. Patients without pathologic involvement of those contralateral SNs are also treated unilaterally. Only when tumor cells are found in a contralateral SN the patient will be treated with standard bilateral ENI. Furthermore, the SUSPECT-2 study has expanded the inclusion criteria to include all patients with HNSCC without evident extension beyond the midline, regardless of the T-classification. It thus strives to further reduce the proportion of patients that undergo bilateral ENI.

### Study objectives and endpoints

#### Objectives

- To investigate the contralateral regional failure rate after radiotherapy (with or without cisplatin-based chemotherapy, cetuximab or immunotherapy) for patients with HNSCC nor crossing the midline, treated with SPECT/CT-guided unilateral ENI.
- To investigate the toxicity of this treatment strategy, and the impact on patients’ quality of life.
- To investigate the prevalence of tumorpositive contralateral sentinel nodes in patients with HNSCC not crossing the midline.

#### Primary endpoint

- Cumulative incidence of contralateral regional metastasis at 1 and 2 years after treatment.

#### Secondary endpoint

b. Early and late radiation-related toxicity
c. Quality of life after treatment

#### Exploratory endpoint

d. Prevalence of (micro/macro) metastasis in contralateral sentinel nodes

## Methods

### Study design

This study has a one-arm, single-center prospective trial design. Patients eligible for this study will undergo SPECT/CT-guided LDM, and based on its findings either unilateral or bilateral ENI will be performed.

### In- and exclusion criteria

#### Inclusion criteria

Eligible for inclusion are patients who are planned for primary radiotherapy (with or without cisplatin-based chemotherapy, cetuximab or immunotherapy) for newly diagnosed primary HNSCC of the oral cavity, oropharynx, larynx (except T1 glottic) or hypopharynx. Eligible disease classification is T1-4 N1 for HPV-positive oropharyngeal cancer, and T1-4 N0-2b for all other tumors (American Joint Committee on Cancer Staging Manual, 8th edition). The tumor needs to be clinically lateralized (not crossing the midline) and histopathologically proven. Patients aged 18 years or older with a performance status of 0–1 (World Health Organization classification) can be included.

#### Exclusion criteria

The main exclusion criteria are clinically tumorpositive contralateral lymph nodes, distant metastatic spread, prior treatment for the current tumor, previous (chemo) radiotherapy or surgery in the head and neck area, and/or recurrent or second primary tumor.

### Pre-treatment evaluation

Standard pretreatment evaluation includes ultrasound of the neck and fine needle aspiration cytology (US-FNAC) performed by a dedicated head and neck radiologist, contrast-enhanced CT scan (and/or MRI), and FDG-PET (Table 1). During US, hypoechogenicity, round shape, border irregularity, a short axis greater than 5 mm, and loss of fatty hilus are considered signs of possible malignancy for which FNAC is performed to confirm or exclude lymph node metastasis. On MRI, a short axis > 10 mm, border irregularity with or without extension in adjacent structures and MRI signs of necrosis are considered signs of possible malignancy. On CT, a short axis > 10 mm, round shape, signs of necrosis, border irregularity with or without extension in adjacent structures are considered signs of possible malignancy. Only in case of a clinically negative neck on one side (N1-2b) or on both sides of the neck (N0), a patient is eligible for inclusion. After the initial work-up, a head and neck surgeon will assess the location and extent of the primary tumor during an investigation under general anesthesia, and will take a biopsy from the primary site.

**Table 1:** Schedule of assessments

	Baseline	During RT	Follow-up					
			1st year^a^				2nd year^a^	3rd-5th year^a^
			12w^a^	6 m^a^	9 m^a^	12 m^a^	every 4 m	every 6 m
Standard of care:								
Physical examination	x	x	x	x	x	x	x	x
Flexible endoscopy	x		x	x	x	x	x	x
Toxicity	x	x	x	x	x	x	x	x
QoL-questionnaire	x		x	x	x	x	until 18th month^a^	
US-FNAC	x		x	x	x	x	when indicated	
CT or MRI^b^	x		x	when indicated				
FDG-PET	x		when indicated					
Investigation under anesthesia	x		when indicated					
RT planning CT	x							
Extra in SUSPECT-2:								
Signed informed consent	x							
Tracer injection and SPECT/CT	x							
Contralateral SNP	only when contralateral drainage is visualized on SPECT/CT							

^a^After end of radiotherapy

^b^According to our institutional guidelines, patients with oral cavity or oropharyngeal tumor are staged by MRI, while laryngeal and hypopharyngeal tumors are staged by CT

Abbreviations: *w* weeks, *m* months, *RT* radiotherapy, *US-FNAC* ultrasound-fine needle aspiration cytology, *SNP* sentinel node procedure

### Interventions

**Fig. 2:**
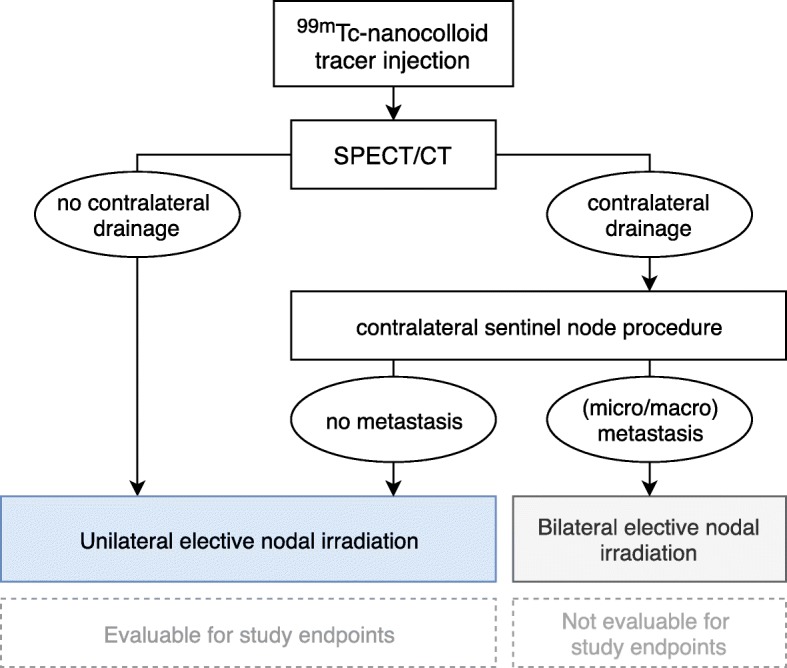
Study flowchart. Flowchart of the SUSPECT-2 study design. Abbreviation: ^99m^Tc: Technetium-99 m

**Fig. 3:**
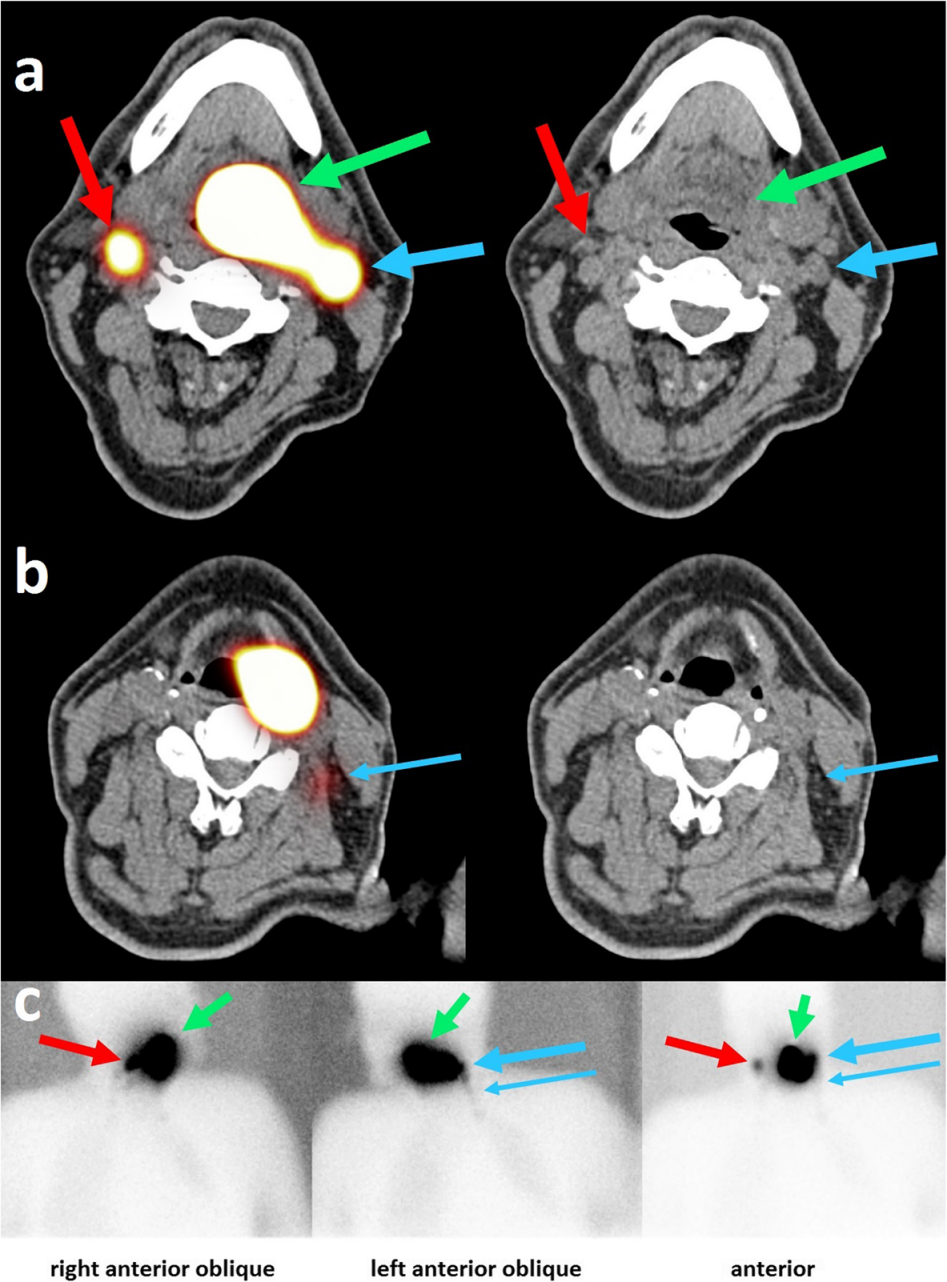
Example of SPECT/CT images. SPECT/CT images (**a**, **b**) and planar lymphoscintigraphy images (**c**) of a 64 year old patient with a T1 N1 base-of-tongue carcinoma. Fused SPECT/CT images are shown on the left panel, CT images are shown on the right panel. On the SPECT/CT images, ^99m^Tc-nanocolloid tracer accumulation is visible, indicating the primary tumor (green arrow), the first ipsilateral draining area in level 2 (large blue arrow), and the decreasing tracer activity down the ipsilateral nodal chain into level 3 (small blue arrow). Furthermore, a contralateral draining area is visible in level 2 (red arrow). In the SUSPECT-2 study, this patient would be a candidate for a contralateral sentinel node procedure on the same day as the lymph drainage mapping. On the CT images, the arrows point to the lymph nodes that are thought to be the anatomical substrates that correlated with tracer accumulation

A flowchart of the study set-up is shown in Fig. 2. On the day of the endoscopy under general anesthesia, the patient will undergo the following procedures:

1. *Injection of radioactive tracer around primary tumor*. Either in the outpatient clinic using flexible endoscopy, or during the endoscopy under general anesthesia, the head and neck surgeon will perform biopsy from the primary tumor site, and will inject a hybrid tracer of indocyanine green (ICG) with (99 m)Tc-nanocolloid in a dose of 80 MBq in a volume of 0.4 cc with 0.05 mg nanocolloid (Nanocoll, Dutch GE Healthcare radiopharmacy, Leiderdorp, The Netherlands). The tracer will be divided in 5 depots, 4 in the mucosa around the primary tumor at 3 mm from macroscopic tumor edges, and one in the tumor itself, according to standard protocol used for surgical procedures of sentinel node biopsy in our institution.
2. *Planar lymphoscintigraphy and SPECT/CT*. After the injection, radioactive tracer migration will be verified using static planar lymphoscintigraphic images followed by SPECT and low-dose CT (SPECT/CT) (40 mAs, 130 Kv) performed at the department of nuclear medicine in radiation treatment position using a personalized radiotherapy mask. Images will be acquired using a dual-head SPECT/CT gamma camera (Symbia T, Siemens, Erlangen, Germany), at 3 ± 1 h after administration, to allow for adequate tracer distribution with maximum sensitivity for contralateral drainage. Planar images are acquired from anterior, left anterior oblique with the head turned to the right, and right anterior oblique with the head turned to the left. SPECT acquisition parameters are 256 × 256 matrix, zoom of 1.0, 2 heads, 180° rotation with 20 views per head (30 s per view). Low-dose CT images are acquired for anatomical correlation with SPECT, and for attenuation correction and scatter correction of SPECT images. For image reading SPECT, CT and fused SPECT/CT are displayed using orthogonal multiplanar reconstruction, maximum intensity projection, and volume rendering. Both a radiation oncologist and nuclear medicine specialist will judge the planar and SPECT/CT images for detection of all sentinel lymph nodes considering their activity and anatomical localization. An example of SPECT/CT images combined with planar lymphoscintigraphy images is shown in Fig. 3. The location of any contralateral draining sentinel nodes is marked on the skin with a marker using a portable gamma camera (Sentinella; Oncovision, Valencia, Spain).

If contralateral lymphatic drainage is visualized on SPECT/CT, the draining contralateral sentinel lymph node(s) are removed by means of:

3. *Contralateral sentinel node procedure*. On the same day, the head and neck surgeon will perform a SNP of the contralateral sentinel lymph node(s), guided by the same hybrid fluorescent-radioactive tracer that was used for the SPECT/CT. The SNP will be performed using multimodal surgical guidance (portable gamma camera, gamma probe and handheld NIR fluorescence camera).

### Pathology examination of removed contralateral SNs

The SNs are fixed in 10% neutral-buffered formalin and three serial sections are cut every 150 μm through the block. At all levels a section is stained with haematoxylin and eosin (H&E) and anti-pan cytokeratin antibody AE1/3. If a metastasis is diagnosed, the size of the metastasis is classified as isolated tumor cells (size ≤0.2 mm), micrometastasis (size > 0.2 mm and ≤ 2 mm), or macrometastasis (size > 2 mm).

### Treatment: radiotherapy regimens

Patients participating in the study will follow the standard preparation procedures for radiotherapy planning, according to institutional guidelines. To the gross tumor volume, a 6 mm isotropic margin will be added to generate the Clinical Target Volume (CTV) of the primary tumor. Elective ipsilateral neck levels will include level I-V in case of node-positive disease and level II-IV in node-negative disease. According to international guidelines, the following tumor site-based exceptions are included [17]:

- In patients with node-negative oral cavity cancer only level I-III will be electively irradiated.
- In patients with node-negative laryngeal and hypopharyngeal cancer, level IV will also be treated electively in case of subglottic extension > 1 cm, transglottic extension and extension into the apex of the piriform sinus.
- In patients with node-negative disease from all sites, the retropharyngeal space will be treated in tumors extending to the posterior wall of the pharynx/larynx and in tumor originating in or extending to the post-cricoid space.
- In patients with node-positive disease, the elective ipsilateral neck levels will also include the adjacent neck level, e.g. in patients with a positive node at the cranial part of level II, the pre-styloid space will also be treated electively.

The inclusion of a contralateral nodal irradiation field will be based on the findings of the LDM using SPECT-CT, and the pathologic examination in case a SNP was performed:

- In case no contralateral drainage was seen on SPECT/CT and no contralateral SNP was performed, no neck levels are irradiated in the contralateral neck.
- In case a contralateral SNP was performed, and pathologic examination found no evidence of metastatic disease, no neck levels are irradiated in the contralateral neck.
- In case a contralateral SNP was performed and pathologic examination revealed macrometastases or micrometastases, contralateral levels II–IV are irradiated according to internationally accepted guidelines [17].
- In case a contralateral SNP was performed but the SN was not identified/detected, the elective irradiation will include the ipsilateral neck plus the contralateral neck level containing the tracer accumulation.

Subsequently, a 3 mm isotropic margin is added to the CTVs to generate the Planning Target Volume (PTV-ENI).

The organs at risk will be delineated according to the internationally accepted guidelines [18] and include the spinal cord, brainstem, cochlea, parotid glands, submandibular glands, thyroid gland, swallowing muscles, oral cavity, supraglottic region, and the larynx.

- PTVprimary tumor = 70 Gy in 35 fractions of 2.0 Gy
- PTVelective nodal irradiation = 54.25 Gy in 35 fractions of 1.55 Gy

Planning will be performed with a Simultaneous Integrated Boost technique using volumetric arc modulated radiotherapy. An accelerated fractionation schedule (6 fractions per week) will be used if radiotherapy is given alone. The 6th fraction shall be given as a second fraction on one of the weekdays with an interval of at least 6 h. When radiotherapy is combined with systemic therapy, conventional fractionation schedule will be applied (5 times per week). All patients receive daily online cone beam CT for positioning imaging.

### Oncologic results

Contralateral regional failures are defined as histopathologically proven contralateral recurrence in lymph node levels which were excluded from the ENI, as defined by the institutional guidelines. The regional control will be assessed during each follow-up visit of patients to the outpatient department. The evaluation of the neck will include US, and FNAC when indicated, every 3 months during the first year after treatment. Since most regional failures occur in the first year after treatment, the analysis for this endpoint will be started one year after the inclusion of the last patient. An overview of standard follow-up assessments is shown in Table 1.

### Assessment of toxicity

Acute (≤90 days after start of treatment) and late toxicity will be evaluated by the radiation oncologist during the weekly visit of patients to our outpatient’s department, and during all follow-up visits afterwards. Toxicity scores will be collected using Common Terminology Criteria for Adverse Events version 5.0 (CTCAE).

### Quality of life assessment

Quality of life (QoL) will be assessed at baseline, and 3, 6, 12, and 18 months after treatment, using the European Organization for Research and Treatment of Cancer Quality-of Life-Questionnaire-C30 (EORTC QLQ-C30) and the European Organization for Research and Treatment of Cancer Quality-of-Life Questionnaire-Head and Neck 35 (EORTC QLQ-HN35). These questionnaires are a standard part of the follow-up visits in our institution and as such represent no additional burden to patients.

### Response evaluation

The response evaluation will be done according to the institutional guidelines 12 weeks after treatment by physical examination of the neck and primary tumor site, including flexible endoscopy. MRI (for oral cavity and oropharyngeal cancer) or contrast-enhanced CT scan (for laryngeal and hypopharyngeal cancer) will be performed to assess the response at the primary site and US-FNAC to assess the nodal state after treatment. In case of doubt about complete response to the primary treatment, FDG-PET followed by examination under general anaesthesia will be done.

### Follow-up

Subsequently, oncologic follow-up visits are scheduled at 6, 9 and 12 months during the first year after RT (including an additional US-FNAC at each visit), every four months in the second year after RT and every 6 months in the third to fifth year after RT (including US-FNAC only when indicated). After five years patients will be discharged from follow-up if there is no evidence of disease. This follow-up schedule is identical to the standard follow-up schedule for HNSCC patients in our institution.

### Sample size estimation

The probability of cRF in carefully selected patients with lateralized HNSCC was estimated to be 2% at 2-years [19–21]. The preliminary results of the proof-of-concept study, the SUSPECT 1, showed that after a median follow-up of 30 months only one patient (2%) developed cRF when treated to one side of the neck. With the inclusion criteria expanded to allow for lateralized T4 tumors, we estimated the rate of cRF to be around 5% or lower. The study was designed to demonstrate a cRF rate ≤ 15%. The limit of 15% is motivated by the fact that an elective irradiation of the neck in case of HNSCC is indicated only when the chance of occult metastasis in the neck exceeds 15% [22–24]. Approximately 90 evaluable patients are required (exact test for a binomial proportion with H0: *p* = 0.15, HA: *p* = 0.05, power = 0.80, α = 0.05, two-sided). Evaluable patients mean those who are treated unilaterally. Patients who are treated bilaterally (patients with contralateral micro−/macrometastases or those with a failed SPECT/CT) will be regarded as not evaluable for the primary and secondary end points and will be replaced by including new evaluable patients. The accrual period of the study is estimated to be 48 months, based on an inclusion rate of at least 2 patients per month.

### Ethics

The study (ClinicalTrials.gov Identifier NCT03968679) will be conducted in accordance with the Declaration of Helsinki and the ICH Harmonized Tripartite Guideline for Good Clinical Practice. The study has been approved by the local research ethics committee (Medical Research Ethics Committee of the Netherlands Cancer Institute/Antoni van Leeuwenhoek, protocol ID: NL68958.031.19). All patients are given oral and written information about the study, and are given sufficient time to consider participating. Written informed consent must be obtained from each patient before inclusion.

### Side studies

The removed lymph nodes will be studied to determine the prevalence of occult metastatic disease in contralateral SNs. Additional information on the technical aspects of peritumoral injections in the outpatient clinic, the contralateral sentinel node procedure and lymphatic drainage patterns will be collected.

## Discussion

(Chemo) radiotherapy for HNSCC is an effective but toxic treatment. A major reduction of the irradiated volume, in the form of unilateral ENI, would facilitate the sparing of (contralateral) organs at risk. If unilateral ENI is proven to be a safe treatment for lateralized tumors, the therapeutic ratio can be improved for a substantial portion of the HNSCC patient population. In the first SUSPECT study, we explored the feasibility of using SPECT/CT as a selection tool for unilateral ENI. Eighty percent of patients treated in the study had unilateral lymphatic drainage and were treated only to the ipsilateral neck, and 20% had bilateral lymphatic drainage and were treated with unilateral ENI plus an elective irradiation dose only to the contralateral neck level where the tracer accumulation was seen. Assuming that metastases are present in only a minority of contralateral sentinel nodes [2, 4, 14–16], this intermediate form of ‘selective bilateral ENI’ might still be an overtreatment in many of those patients.

This study aims not only to validate the findings of the initial SUSPECT study, but also to provide insight into the prevalence of metastasis in contralateral SNs. To ensure continuity with the initial SUSPECT study, and to allow for the highest sensitivity for contralateral drainage, also in bulky tumors, the radioactive tracer is injected both peri- and intratumoral. If the prevalence of metastasis in contralateral SNs is sufficiently low (< 10%), unilateral ENI could in the nearby future be applied in selected patients without the need for lymph drainage mapping. When the results of this follow-up study reinforce the promising oncologic outcomes of the initial SUSPECT study, the head and neck radiation oncology community will be encouraged to expand the indications for unilateral ENI in HNSCC.

### Current status

The study received approval from the local research ethics committee and is presently ongoing. It included its first patient in July 2019.

## Data Availability

Data sharing is not applicable to this article as no datasets were generated or analysed during the current study.

## Abbreviations

cRF: contralateral regional failure
CTV: Clinical target volume
ENI: Elective nodal irradiation
Gy: gray
HNSCC: Head and neck squamous cell carcinoma
ICG: Indocyanine green
LDM: Lymph drainage mapping
PTV: Planning target volume
QoL: Quality of life
SN: Sentinel node
SNP: Sentinel node procedure
SPECT/CT: Single Photon Emission Computed Tomography/Computed Tomography
US-FNAC: Ultrasound – fine needle aspiration cytology
